# The Minds of God(s) and Humans: Differences in Mind Perception in Fiji and North America

**DOI:** 10.1111/cogs.12703

**Published:** 2019-01-07

**Authors:** Aiyana K. Willard, Rita A. McNamara

**Affiliations:** ^1^ Centre for Culture and Evolution Brunel University London; ^2^ Institute of Cognitive and Evolutionary Anthropology University of Oxford; ^3^ School of Psychology Victoria University of Wellington

**Keywords:** Mind perception, Opacity of mind, Mind of god, Religion, Fiji, Cross‐cultural research

## Abstract

Previous research suggests that how people conceive of minds depends on the culture in which they live, both in determining how they interact with other human minds and how they infer the unseen minds of gods. We use exploratory factor analysis to compare how people from different societies with distinct models of human minds and different religious traditions perceive the minds of humans and gods. In two North American samples (American adults, *N *=* *186; Canadian students, *N *=* *202), we replicated a previously found two‐factor agency/experience structure for both human and divine minds, but in Fijian samples (Indigenous iTaukei Fijians, *N *=* *77; Fijians of Indian descent, *N *=* *214; total *N *=* *679) we found a three‐factor structure, with the additional containing items related to social relationships. Further, Fijians’ responses revealed a different three‐factor structure for human minds and gods’ minds. We used these factors as dimensions in the conception of minds to predict (a) expectations about human and divine tendencies towards punishment and reward; and (b) conception of gods as more embodied (an extension of experience) or more able to know people's thoughts (an extension of agency). We found variation in how these factors predict conceptions of agents across groups, indicating further theory is needed to explain how culturally generated concepts of mind lead to other sorts of social inferences. We conclude that mind perception is shaped by culturally defined social expectations and recommend further work in different cultural contexts to examine the interplay between culture and social cognition.

## 1 Introduction

Humans have a remarkable ability to conceive of and reason about minds. Starting in infancy, we are able to decode observable physical cues—such as facial expressions, body language, and gaze—to understand the unobservable mental states of those around us (e.g., Baron‐Cohen, 1995; Ekman, 1993; Johnson, Slaughter, & Carey, 1998; Meltzoff, 1995; Woodward, 1998). These abilities, and the ability to socially learn and transmit knowledge that comes with them, are part of the foundation of our remarkable ability to create the myriad of different cultures we live in (Chudek & Henrich, 2011; Chudek, Zhao, & Henrich, 2013; Henrich, 2015). These cultural differences can, in turn, affect how we conceive of and reason about minds (e.g., ojalehto, Medin, & García, 2017a,b). Some of the most striking examples of how culture can influence mind concepts are in how we understand the minds of gods and spirits (Astuti & Harris, 2008; D'Andrade, 1987; Knight, 2008; Knight, Sousa, Barrett, & Atran, 2004; Lane, Wellman, & Evans, 2010). Humans readily apply mental state reasoning to a wide variety of non‐human, non‐mentalistic, and even inanimate phenomena to create an assortment of religious and other supernatural beliefs (Guthrie, 1993; Heiphetz, Lane, Waytz, & Young, 2015; Waytz, Epley, & Cacioppo, 2010; Waytz et al., 2010; Willard & Norenzayan, 2013). Along with the ubiquity of these supernatural minds in human cultures, there appears to be substantial variation in the types of supernatural minds people create (see Purzycki & Sosis, 2011).

How humans conceptualize supernatural agents’ minds provides a unique window into the broader processes underlying how we think about minds in general. Beliefs about the content of both human and supernatural minds rely on inference—no matter how much one might feel like we can read other people like a book, we can never have direct access to what is going on in someone else's head. Instead, we rely upon indirect clues like facial expressions, behaviors, and words. Unlike human minds, supernatural agent minds do not offer these observable cues to guide how we understand them; our conceptions of supernatural minds are built largely from a cultural understanding of minds applied to otherwise ambiguous phenomena that may not be obviously relevant for mental state reasoning (Guthrie, 1993, 1996).

Though people generally think of supernatural minds as having different characteristics than human minds, our understanding of human minds forms the basis for our understanding of supernatural minds. Across French, Italian, Australian, and Chinese samples, participants see gods as having more powerful minds than humans (Demoulin, Saroglou, & Van Pachterbeke, 2008; Haslam, Kashima, Loughnan, Shi, & Suitner, 2008). In North America, people describe God as possessing highly agentic qualities, such as the ability to think and remember. On the other hand, North Americans also describe God as having few of the experiential qualities—such as the ability to feel hunger and pain—that are typically applied to humans (Gray, Gray, & Wegner, 2007; Gray & Wegner, 2010). Though some of this work has been done across different societies (e.g., Haslam et al., 2008), it does not represent enough diversity to know if these tendencies are consistent across different groups. Cultural differences may substantially change how we see supernatural minds, even within a single well‐studied religious group like Christianity.

In this paper, we examined some of the findings of the mind perception literature across societies with different religious traditions and distinct normative stances on how to understand minds. We looked at mind perception—or how people conceive of minds—in two North Americans samples and two Fijian samples. The North American samples consisted of a sample of Canadian university students and a sample of American adults from Amazon's Mechanical Turk. In addition to being the type of sample used in much of the existing research (Henrich, Heine, & Norenzayan, 2010), both North American samples live in a cultural setting where the mind is often claimed as the ultimate source of all behavior, and therefore the best source of information for interpreting others actions.

Our two Fijian samples were samples of Indigenous iTaukei Fijians and Indo‐Fijians. iTaukei Fijians typically follow very different informal rules for understanding and interpreting other people, preferring to avoid intruding into the private mental space of others by talking about the content of others minds and instead focusing on externally visible actions (McNamara, Willard, Norenzayan, & Henrich, 2019). Indo‐Fijians live in the same broad societal setting as iTaukei Fijians, yet typically hold to a third approach to minds, focusing more on internal mental states than the iTaukei Fijians, while still emphasizing group affiliations and family ties in their perceptions of people.

Along with these societal‐level, normative differences in how one should think about minds, we also sampled across different kinds of religious beliefs. Our North American samples included participants from a mostly Christian background, along with a sizable number of non‐religious participants. Our Indigenous iTaukei Fijian sample was devoutly Protestant Christian, though many participants also routinely reference beliefs in pre‐Christian ancestor spirits. Our Indo‐Fijian sample was predominantly Hindu, with a subset of Muslim participants. Unlike the Abrahamic God of Christianity and Islam, Hindu gods are seen as having physical needs and often a physical presence. They are ritualistically fed, bathed, and clothed (Fuller, 2004). These diverse samples allowed us to examine how conception of human minds feeds into conception of supernatural minds, and how wider societal norms about how to approach the problem of other minds influence beliefs about other humans and supernatural beings.

We offer a cross‐cultural comparison of several different aspects of the current dimensions of mind perception literature:

1. The factor structure of minds in different groups (e.g., Gray et al., 2007; Weisman, Dweck, & Markman, 2017);
2. How groups conceive of the minds of humans and gods in relation to these factors (e.g., Gray & Wegner, 2010);
3. How these different factors relate to morally relevant characteristics of reward and punishment (Gray & Wegner, 2011);
4. How these factors relate to the physicality and supernatural mindreading abilities of the target mind (in this case gods; e.g., Gray, Knobe, Sheskin, Bloom, & Barrett, 2011).

## 2 Cultural differences in conceiving of and reasoning about minds

Much of the existing research on mind perception starts from the premise that the mind is the origin of all behavior, and that individuals are largely the product of their internal mental processes (Henrich et al., 2010). This approach to mind perception as the core of person perception is often presumed to apply universally, but it may in fact be a notion that grows out of uniquely Western cultural traditions (see Lillard, 1997, 1998). Some aspects of mind perception such as reasoning about false beliefs (e.g., Barrett et al., 2013; Callaghan et al., 2005; Lane et al., 2010; Liu, Wellman, Tardif, & Sabbagh, 2008; Wellman, Cross, & Watson, 2001), and presumably early arising behaviors like understanding intention (e.g., Meltzoff, 1995; Woodward, 1998) and gaze direction (D'Entremont, Hains, & Muir, 1997; Johnson et al., 1998), are likely candidates for consistency across cultures. Beyond this, a substantial amount of variation in how societies conceptualize almost every aspect of minds and mental state reasoning has been recorded in the ethnographic record (see Lillard, 1997, 1998).

There are some existing psychological studies that show that both culture and religious beliefs can affect how individuals reason about minds. Work with the Ngöbe people of Panama has found that the Ngöbe use ecological and social relationships, rather than animacy and consciousness, as a basis for agency (ojalehto et al., 2017a,b). This is illustrated by the finding that the Ngöbe include plants and abiotic entities in their category of things that have agency and a capacity for intentional actions along with animals or humans. This differs from the more often sampled groups in North America, who include only animals and humans in the category of things that have agency. Other work has shown that religious beliefs in Madagascar impact how people think about the life of the mind after death (Astuti & Harris, 2008). This latter work broadly replicates how religion impacts beliefs about the continuation of the mind after death in North America (Bering & Bjorklund, 2004; Bering, McLeod, & Shackelford, 2005; also see Heiphetz et al., 2015; Lane et al., 2010).

Another approach to minds that is of particular note is described in ethnographic accounts from communities around the Pacific, including Fiji, and in parts of Mayan Mexico. These accounts suggest that people in these societies hold to a belief that one can never truly understand, and therefore should not try to infer, the minds of others (Duranti, 2015; Groark, 2013; Hollan, 2012; Hollan & Throop, 2008; Lutz, 1988; Robbins & Rumsey, 2008). This belief that minds cannot be known is called “Opacity of Mind.” People in societies that hold Opacity of Mind beliefs state that reference to the contents of another's mind is impolite or impossible, and many show a preference for interpreting people's actions based upon observable behaviors and explicit statements rather than unseen intentions or motivations.

Opacity beliefs do seem to have an impact on how people in these societies reason about minds. Developmental research has found that in societies with Opacity of Mind, children pass the false belief task at older ages than non‐Opacity countries (Callaghan et al., 2005; Dixson, Komugabe‐Dixson, Dixson, & Low, 2017; Knight et al., 2004; Mayer & Trauble, 2012). These beliefs also impact moral reasoning. In societies with Opacity of Mind, if an action produced some kind of wrong or harm, then intentionality will not necessarily mitigate people's negative judgments—accidentally taking a bag that was not yours will be judged similarly to an intentional stealing and more harshly than a failing attempt to steal (McNamara et al., 2019). The specific mechanisms and pathways behind the relationship between these Opacity of Mind beliefs and cognitive development are as yet unknown. One possibility is that a cultural environment that dissuades mental state reasoning may reduce the overall amount of information about mental states available to learners and slow the process of learning about mentalizing processes and how to apply them to assess behavior.

## 3 Dimensions of human and supernatural mind perception: Agency and experience

Previous research has found that North Americans conceive of minds with two factors: agency and experience (Gray et al., 2007; for an alternative factor structure, see Weisman et al., 2017). Agency consists of things such as thoughts, memories, and morality, where experience is made up of emotions and sensations like anger, hunger, and fear. Within this research, North Americans have been shown to rate God's mind as made up almost entirely of agentic capacities (e.g., intelligence, morality, consciousness), with few or no capacities that relate to emotion or sense‐based experiences (Gray & Wegner, 2010; Gray et al., 2007). God's mind is seen as dispassionate and all knowing, yet moral, even among children (Heiphetz et al., 2015; Lane et al., 2010). God does not get hungry or feel pain, nor does God feel pleasure or embarrassment. This view of God is probably unsurprising to most Western readers; it is a conception of God that is found across Abrahamic traditions and is common in the Western world, but may be much less prevalent in other parts of the world (see Purzycki & Sosis, 2011).

A survey of religious traditions suggests that the variation in beliefs about gods and gods’ minds around the world is immense (Purzycki & Sosis, 2011). Representations of gods and supernatural agents across cultures span everything from animals (e.g., Coyote in indigenous American traditions), elements (e.g., Agni in Vedic Hinduism), and human‐like beings with physical bodies (e.g., Zeus in Greek mythology), to non‐corporeal spirits and omniscient minds (e.g., Abrahamic God). What these different types of gods and other sorts of supernatural agents care about differs as well. The spirit‐masters of the Tyva Republic have special knowledge and care about the human world, but only if you are physically near them (Purzycki, 2013). The Japanese Kami are thought of more like essences than minds, but they can still help you with a problem that falls within their domain or punish you when you treat them wrongly (see Kitagawa, 1987). Within Hinduism alone, the variation in how believers conceive of gods, their minds, their bodies, and how they exist in relation to humans is too extensive to review in any concise way, but in many cases gods are seen as having a physical presence in the world (see Fuller, 2004).

Even with these differences, all of these gods have something we can identify as a mind or mentalistic abilities (Purzycki & Sosis, 2011). This variety of minds offers us a rich array of the different ways humans are capable of conceiving of minds, and may help us to better understand some of the boundaries to how people are able to understand minds and how cultural differences can inform these processes.

## 4 Overview of samples: Mind and religion in Fiji and North America

### 4.1 Fijian samples and religious beliefs

Fiji has two major ethnic groups: the indigenous iTaukei Fijian population and a diaspora population from India (Indo‐Fijians). iTaukei Fijians consider Christianity as a core aspect of their identity, though many still hold some of their traditional religious beliefs, especially in the more traditional villages. These beliefs focus around ritual offerings of yaqona (kava), a mildly narcotic traditional beverage, offered to the Kalou‐vu (Katz, 1999; Shaver, 2014). The Kalou‐vu are not gods, but the deified ancestral progenitors of the clans that form the backbone of traditional Fijian social hierarchies. The Kalou‐vu are believed to care about traditional norms and can affect the health and fortune of those who deviate from traditions, but they can also be called on for traditional medicine among those who lead a proper traditional lifestyle (Katz, 1999; McNamara & Henrich, 2017a). They are not visually depicted, though some folktales about particularly important Kalou‐vu do include physical descriptions. These physical characteristics combine human and animal traits. The major Christian denominations in our sample were Wesleyan Methodist and the Assemblies of God Pentecostals, though several other Christian denominations exist around Fiji.

Most Indo‐Fijians are Hindus or Muslims, with a small minority of Sikhs and a growing minority of Christians. This population was brought to Fiji by the British as indentured laborers between 1879 and 1912 (Gillion, 1962). Currently, Indo‐Fijians make up 37% of the population of Fiji, though until recently this percentage was much higher with many Indo‐Fijians recently leaving Fiji (Voigt‐Graf, 2008). The Indo‐Fijians live primarily in and around Fiji's cities and towns and work either as sugar cane farmers or in urban centers. Though the Fijian Hindus believe in multiple gods, they believe that all of their deities are just different aspects of a single God (Willard, 2017). Our Hindu Indo‐Fijian participants have perhaps the most human‐like pantheon of all of our samples, with gods that have lived among humans, require food and feeding, clothes and various other comforts not offered to the Abrahamic God of the other samples.

Both the iTaukei and Indo‐Fijians live in highly collectivistic communities. They have strong family ties and frequently rely on their social networks for help in times of need (Gervais, 2013; Kelly, 2001; Kline, Boyd, & Henrich, 2013; Lal, 1992; McNamara & Henrich, 2017b; Shaver, 2014). Further, both the Indo and iTaukei communities have more hierarchically structured social roles than is normally experienced in North America. Previous work with Indians living in India suggest that this hierarchical power differential results in different patterns of emotional intelligence—a capacity that is intimately tied with how people understand minds—with items about impression management and making a good impression being more emphasized in India than in a comparison sample of Germans (Sharma, Deller, Biswal, & Mandal, 2009).

The variety of religious groups in the Fijian samples gives us a window into how different religions may shape different conceptions of Gods’ minds across different groups within the same country. The iTaukei participants allow us to look at how different cultures can influence the conception of the Christian God's mind. The view of the Christian God extrapolated from the standard North American sample may be largely inconsistent with how many Christians think about their deity throughout the world. These differences in social relationships and religious beliefs make it plausible that our samples will differ in how they think about the minds of other people as well as the minds of gods.

### 4.2 North American samples and religious belief

We collected two North American samples for this research: students from a Canadian university psychology subject pool and an American adult sample collected online from Amazon's Mechanical Turk. North America contains some of the most individualistic societies in the world today (Markus & Kitayama, 1991; Triandis, 1995), making them very unlike the Fijian samples on this front. Further, family structure tends to be more nuclear and does not include as much hierarchy or interaction from extended family (Hofstede, 1980). These samples are also far less religious on average. The Canadian University sample was collected in Vancouver Canada. Vancouver is ethnically and religiously diverse, with a substantial Asian‐Canadian population (44%).

These samples are fairly standard samples used in this type of psychological research and much of the previous research on mind perception and the dimensions of mind perception have used one of these two types of samples. Psychological subject pools have been claimed as problematic and often do not generalize to the population at large (Hanel & Vione, 2016), yet they are still used at a high level in psychological research. Online samples such as Mechanical Turk samples have been employed as a way to partially correct this problem (Buhrmester, Kwang, & Gosling, 2011; Goodman, Cryder, & Cheema, 2012), but they cannot account for cultural variance in psychological variables outside of users of these services, and they are extremely limited in terms of countries from which samples can be collected. Both Canada and the United States are predominantly Protestant Christian countries with a growing number of non‐religious (Pew, 2012).

## 5 Current research

The primary goal of this research was to evaluate how our Fijian samples conceive of the minds of humans and gods and compare this to the conceptions found in our North American samples (see Gray et al., 2007). We approached this goal in three parts. First, we used exploratory factor analyses to assess if Fijians and North Americans use similar dimensions of mind perception to conceive of the minds of humans and gods differently. We expected to replicate the agency/experience two‐factor structure in both North American samples for both humans and God's mind, but we had no strong prior predictions about the factor structure found with the Fijian samples. Because our Indigenous iTaukei Fijian sample exhibits strong societal‐level prohibitions against mental state inference, we initially expected our iTaukei Fijian sample to see mental dimensions as less different from one another than the other two cultural groups, resulting in more neutral ratings of all dimensions. We further expected that, to the extent that iTaukei Fijian participants do differentiate mental dimensions, both Fijian samples would be more focused on social or group relevant mental states rather than more individualistic ones, as they are particularly relevant in these hierarchically structured and collectivist societies. This research was exploratory and should be interpreted as such.

Second, we looked at how the ratings for these factors related to beliefs about the tendency of humans and gods to reward and punish others. Reward and punishment are one of the primary ways gods are believed to interact with humans and form much of the foundation for the moral role of these agents across societies (Johnson, 2015; Norenzayan, 2013; Norenzayan et al., 2016; Purzycki et al., 2016, 2018). Similarly, social punishment by other humans is one of the foundations for enforcing normative behavior in cooperative societies (Boyd, Gintis, Bowles, & Richerson, 2003; Fehr & Fischbacher, 2003; Fehr & Gachter, 2005; Henrich et al., 2006). Reward and punishment give us two morally relevant characteristics that are applicable to both humans and gods, and that should be related to the mental dimensions (Gray & Wegner, 2011; Gray, Young, & Waytz, 2012). North Americans see intentional harm as much more punishment worthy than unintentional harm (Cushman, 2008; McNamara et al., 2019). The more agency given to people, the more responsible they are for their own actions (Gray & Wegner, 2011), and the more capable they are of enacting moral punishment on others (Gray & Wegner, 2010). Researchers have gone so far as to say that mind perception abilities are the essence of moral reasoning (Gray et al., 2012). Despite this, how intention is used to determine the consequences of actions is variable across cultures (Barrett et al., 2016; McNamara et al., 2019).

Opacity of Mind beliefs suggest that people should be rewarded or punished based on the outcome of their actions regardless of intention or emotional state. If explicit Opacity of Mind beliefs fully dictate how our iTaukei Fijian participants determine the consequences of actions, then mental states should be less related to the inclination to reward and punish in this sample than in the other samples. In what we call the Strong Opacity prediction, if reward and punishment are to be mechanistically distributed based on actions alone, then how mentalistic an agent is should be completely unrelated to ratings of tendencies to reward or punish others. Alternatively, Opacity norms may be more about politeness than an actual restriction on using mental states—iTaukei Fijians may see the discussion of others’ mental states and internal motivations as an invasion of privacy but still use these concepts when thinking about and understanding the actions of others. In this Light Opacity prediction, we should find that iTaukei Fijians’ ratings of mental states do predict expectations about reward and punishment tendencies. Whether these relationships between mental state ratings and behavioral expectations fit the same cultural structure as in North Americans or even other Fijians is an open question. Given that our Indo‐Fijian participants are often more likely to openly discuss mental states like North Americans but also more likely to focus on social relationships like iTaukei Fijians, we expect the relationships between their mental state ratings and behavioral expectations to fall somewhere between iTaukei and North American results.

Finally, we used questions asking if God has a physical body, and if God can know a person's thoughts to test whether the factor ratings of God's mind relate to how physical/mentalistic a supernatural agent is thought to be. These ratings are especially likely to pick up on underlying differences in dimensions of agency vs. experience. Previous research has found that directing participants’ attention to the physical body in photographs of people causes higher ratings of experience and lower ratings of agency for the photographed people (K. Gray et al., 2011). This work also found that people who are evaluated on intelligence and efficiency are given lower ratings of experience than those evaluated on attractiveness. Thinking about a person's physicality made participant raters see their targets as more emotional and as experiencing more sensations like pain and hunger, further supporting this division. If this is generalizable across minds and cultures, gods with physical bodies (such as Hindu gods) should be rated as having more ability to experience emotional and physical sensations.

## 6 Methods

### 6.1 Participants

Four samples were collected from Canada, the United States, and Fiji (basic demographics listed in Table 1). The iTaukei Fijian sample came from a small traditional village on the Yasawa Islands, where they still live largely based on subsistence horticulture and fishing, with little access to electricity and other modern amenities. All of the iTaukei Fijian sample was Christian. The Indo‐Fijian sample consisted of Hindus and Muslims primarily living in the Lovu area (a set of villages outside the town of Lautoka). Some additional Muslim participants were recruited around the nearby towns of Nadi and Ba. Canadian students were collected from the University subject pool and received class credit for their participation. Of the 35 in the “other religion” category, 10 were Buddhist, 4 were Hindu, 4 were Jewish, 3 were Muslim, 2 were Sikh, 2 were Baha'i, and the remainder were other religions. American adults were recruited through Amazon's Mechanical Turk and paid $1.50 for their time.

**Table 1:** Demographics

	*N*	Gender	*M*_age_ (*SD*)	Religion
Canadian university students	202	169 female	19.70 (2.16)	92 religious (57 Christian, 35 other) 110 not religious
American adults from MTurk	186	96 female, 1 other	33.90 (12.39)	91 religious (86 Christian, 5 other) 95 not religious
Indo‐Fijians	214	120 female	38.13 (15.30)	136 Hindu 78 Muslim
iTaukei Fijians	77	48 female	42.95 (15.19)	All Christian

### 6.2 Materials

The questionnaires consisted of a list of mentalistic capacities and basic demographic questions. Participants were asked to rate “most people” and “God” on a set of capacities. Questions about God were asked in comparison to humans (e.g., “How good are most people at remembering things?”, “Compared to most people, how good is God at remembering things?”). This was necessary in the Fijian populations as they tend to rate many questions about God at the maximum value when they are asked in the abstract. Further, comparing God to humans in the questions themselves gives a concrete baseline for rating of God across cultures that accounts for differences in perceptions of human minds. These questions were based on the capacities used in Gray et al. (2007). Some of the terms were changed and simplified so they would be easier to translate and be understood in the Fijian samples (see Appendix S1 for complete list). All questions were asked on a 5‐point scale from −2 to 2, where 0 was neutral, the positive numbers were good at/high in the capacity, and the negative numbers were bad at/low in the capacity.

Fijian versions of the questionnaire were presented in three randomly generated orders, while students and MTurk samples answered questions in a new random order for each participant, as generated by our online survey software (SurveyMonkey.com). Reward and punishment questions that were asked in relation to behavior (i.e., rewarding good behavior or punishing bad behavior) and measured on the same scale (−2 to 2) were included as part of this questionnaire (“How much do most people reward/punish other people for their good/bad behavior,” “Compared to most people, how much does God reward/punish people for their good/bad behavior?”). Participants were asked if God had a body and if God could know your inner thoughts. These questions were both presented as binary yes or no questions and were not asked about humans.

### 6.3 Procedures

iTaukei materials were translated into Standard Fijian and then back translated into English by university trained iTaukei Fijian research assistants who were from another part of the archipelago. The questionnaires were similarly translated and back translated into Fiji‐Hindi for the Indo‐Fijian population. Indo‐Fijians in these areas are mostly bilingual, speaking both English and Fiji‐Hindi. Some participants spoke only Fiji‐Hindi or primarily spoke English. Additionally, some terms were more common in one or the other language. Thus, all questions were asked in both languages.

The Indo‐Fijians and the Canadian sample answered both the human and God questions in one session. Due to the constraints of the iTaukei communities in Yasawa, the iTaukei participants answered these questions in separate sessions. For both the Indo‐Fijians and iTaukei, questions were read out loud by a research assistant in participants’ homes. Visual scales were used and answers were given by pointing to a point on the scale. The Mturk sample completed the questionnaire online. The Canadian students completed the task on a computer in a laboratory setting. A subset of the Canadian student sample (*N *=* *47) was interviewed in the laboratory by a Canadian research assistant in the same manner as the Fijian samples to check for differences between collection methodologies. No significant differences were found on the average item ratings between these groups (*F*(1,200) = 0.55, *p *=* *.46); therefore, they were combined into a single sample. In addition to answering questions about the Christian God, the iTaukei participants completed the questionnaire for the Kalou‐vu.

## 7 Results

### 7.1 Factor analyses

We used an exploratory factor analysis to assess how these groups structure mental capacities. The samples were combined into two groups: North Americans (Student and MTurk) and Fijians (iTaukei and Indo). Analyses were performed within these groups. Human (see Table 2) and God questions (see Table 3) were factored separately for each group. The Fijian sample combines two distinct ethnic groups and three religious groups. Potential problems created by this are addressed in each case. A parallel scree plot analysis was conducted to determine the appropriate number of factors in each sample for all factor analyses, and samples were factored accordingly. Maximum likelihood estimation and oblimin rotations, to allow for correlations between factors, were used for all factor analyses. In the factor tables, loading higher than 0.6 are in dark gray and the items used to form each factor in subsequent analyses are in medium gray (items with loadings of higher than 0.30). This lower threshold was chosen because of the substantially weaker relationships in the Fijian data. The increased noise in the Fijian data could have been caused by a lack of familiarity with questionnaires and Likert scales or represent a weaker association between capacities in this sample. Positive cross‐loadings larger than 0.30 are light gray and those below 0.30 are left white.

**Table 2:** Exploratory factor analysis results for students vs. Fijians on human targets

North America—Human			Fiji—Human			
Item	Exp.	Agency	Item	Exp.	Agency‐Self	Agency‐Other
Anger	0.76	−0.12	Pain	0.64	−0.04	−0.11
Fear	0.74	−0.05	Fear	0.58	−0.02	−0.01
Embarrassed	0.66	−0.01	Anger	0.56	0.08	0.07
Pain	0.66	0.10	Desires material	0.49	−0.13	0.08
Desires material	0.65	−0.08	Hunger	0.43	−0.08	0.20
Desire attention	0.60	0.02	Desire attention	0.40	−0.16	0.11
Hope/wish	0.60	0.06	Embarrassed	0.40	0.30	−0.24
Pride	0.57	0.04	Pride	0.36	−0.10	0.09
Hunger	0.55	0.13	Hope/wish	0.36	0.07	−0.03
Pleasure	0.51	0.35	Aware	0.00	0.76	0.03
Others feel	−0.06	0.66	Know right/wrong	0.01	0.65	−0.03
Thinking	−0.04	0.65	Others feelings	−0.19	0.45	0.11
Self‐control	−0.21	0.64	Remembering	−0.15	0.33	0.13
Remember	0.06	0.64	Pleasure	0.23	0.27	0.11
Know right/wrong	0.00	0.59	Joy	0.22	0.24	0.07
Plans/goals	0.03	0.56	Plans/goals	0.07	0.04	0.70
Aware	0.17	0.50	Thinking	−0.27	0.08	0.40
Joy	0.35	0.45	Self‐control	−0.17	0.26	0.29

Factor Correlations and Variance			Factor Correlations and Variance			
	E	A		E	A‐S	A‐O
Experience	(0.94)		Experience	(0.88)		
Agency	0.37	(0.91)	Agency self	−0.20	(0.87)	
			Agency other	−0.21	0.21	(0.78)
Prop. Variance	0.24	0.17	Prop. Variance	0.13	0.10	0.05

Note

Loadings of 0.60 or higher are highlighted in dark gray; loadings of 0.30 or higher are highlighted in medium gray; and cross‐loadings of 0.30 or higher are highlighted in light gray.

**Table 3:** Exploratory factor analysis results for students vs. Fijians on God as the target

North America—God			Fiji—God			
Item	Exp.	Agency	Item	Exp.‐Social	Exp.‐Basic	Agency
Desires material	0.73	−0.17	Hope/wish	0.59	0.02	−0.01
Embarrassed	0.73	−0.05	Embarrassed	0.56	0.04	−0.09
Hunger	0.73	−0.05	Anger	0.50	0.05	−0.01
Fear	0.71	−0.01	Pain	0.44	−0.11	0.07
Hope/wish	0.47	0.36	Pleasure	0.41	−0.06	0.21
Pride	0.43	0.32	Pride	0.39	0.16	0.16
Pain	0.40	0.40	Desire attention	0.36	0.02	0.33
Anger	0.36	0.34	Desires material	−0.06	0.86	0.02
Desire attention	0.32	0.31	Fear	0.01	0.69	0.07
Others feelings	−0.06	0.92	Hunger	0.22	0.59	−0.18
Thinking	−0.06	0.91	Aware	0.03	0.04	0.71
Know right/wrong	−0.11	0.90	Other feel	−0.1	−0.07	0.71
Self control	−0.05	0.88	Remembering	−0.02	0.04	0.60
Aware	−0.01	0.87	Thinking	−0.01	0.07	0.66
Remember	−0.06	0.87	Know right/wrong	0.09	−0.04	0.59
Plans/goals	−0.02	0.83	Plans/goals	−0.01	0.07	0.54
Joy	0.15	0.81	Joy	0.15	−0.06	0.49
Pleasure	0.30	0.68	Self control	0.21	0.08	0.31

Factor Correlations and Variance			Factor Correlations and Variance			
	E	A		E‐S	E‐B	A
Experience	(0.98)		Exp. S	(0.88)		
Agency	0.07	(0.91)	Exp. B	0.25	(0.95)	
			Agency	0.21	−0.16	(0.87)
Prop. Variance	0.16	0.37	Prop. Variance	0.10	0.09	0.17

Note

Loadings of 0.60 or higher are highlighted in dark gray; loadings of 0.30 or higher are highlighted in medium gray; and cross‐loadings of 0.30 or higher are highlighted in light gray.

Based on the scree plot analysis, a two‐factor structure was found for the human mind in the North American sample (RMSEA = 0.052, 90% CI: 0.033–0.064; Table 2). These factors largely follow those found in Gray et al. (2007) and are labeled “agency” and “experience” accordingly. Unlike Gray et al. (2007), we found that joy loaded more strongly onto the agency factor than the experience factor, though both pleasure and joy had cross‐loading above our cutoff point of 0.3.

We found three factors for human minds in the Fijian sample^1^ (RMSEA = 0.056, 90% CI: 0.042–0.066). One of these factors corresponds broadly to the experience factor found in the North American sample, but the agency factor was further divided into two separate factors: one dealing with primarily other‐related mental capacities (e.g., morality, awareness, understanding others’ feelings), and one dealing with primarily self‐related mental capacities (making plans and having goals, thinking). We have labeled these two factors agency‐other and agency‐self, respectively. Pleasure, joy, and self‐control were not included in the factors because of their weak loadings in this sample. Excluding the iTaukei sample does not change the number of factors or the items that load on each factor (see Appendix S1). The sample of iTaukei participants is too small to reliably predict factors and thus was not run as a separate sample. This sample consists of the entire adult population willing to participate in our study within the villages sampled. It was therefore not possible to collect a larger sample.

In analyses of the God questions, we find that the North American data divided into the same two factors as the human mind questions, corresponding to agency and experience (RMSEA = 0.078, 90% CI: 0.062–0.088; Table 3)—with the exception of pleasure moving from the experience to the agency factor. However, this data did not factor as cleanly as for human targets and contains some substantial cross‐loadings between factors.

The Fijian sample presents a different challenge. Our sample includes ratings of three different gods, with only the Hindus having a large enough sample to run independently. The factor analysis presented here combines both the Indo‐Fijian groups (Hindu and Muslim) and iTaukei ratings of the Christian God. The Kalou‐vu were excluded because the Christian God and the Kalou‐vu were rated by the same people, creating non‐independent data points.

The Fijians again show three factors (RMSEA: 0.065, 90% CI: 0.051–0.074), but these factors are not the same as the factors found for human minds. Experience was split into two factors, rather than agency. The first experience factor, which we have called experience‐social, has to do with the more social emotions and other‐referencing elements of the experience factor, such as pride and embarrassment. This may similarly come out of the more relational aspects of Fijian culture. The second experience factor, which we have called experience‐basic, contains the more base needs and responses of a human‐like agent: desire for material possessions, fear, and hunger. It could be argued that this second factor contains items that reflect things not relevant to gods, but it is not clear this is the case, at least for Hindus. Indo‐Fijian Hindus regularly engage in ritual feeding and clothing of their deities, and bring them money and gifts (Kelly, 1987; Willard, 2017). It may be that this second experience factor comes out of the data entirely due to the Hindus in the sample, but additional analyses do not support this interpretation either. Factor analysis using either just the Hindus or a combination of Muslims and Christians does not change the factors in any substantial way (see Appendix S1). As with the North American samples, there were sizeable cross‐loadings in the Fijian samples.

**Figure 1:**
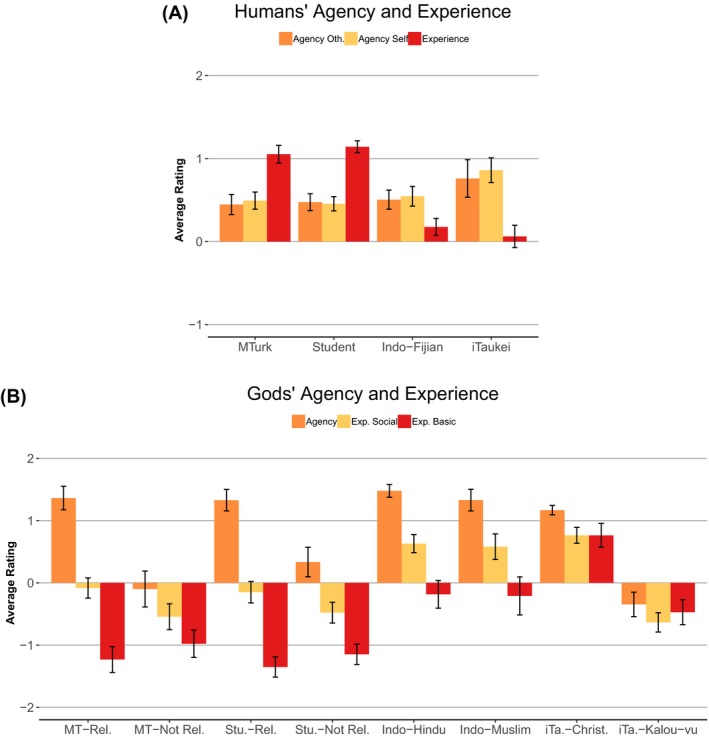
Differences in models of human minds (A) and gods minds (B) across MTurk, students, Indo‐Fijian, and iTaukei samples. Error bars are 95% confidence intervals. Rel., religious; Not Rel., not religious.

Despite the difference in the North American and Fijian samples, all divisions can be collapsed into the same two broad categories. However, these two categories may not fully represent the complexities of how different cultural groups think about minds. For the purpose of comparison, we used the divisions of agency and experience as they are presented in the Fijian sample in Fig. 1 (see Appendix S1 for full analysis of these differences).

For both humans and gods, there was substantial variation across samples in the average ratings for each factor. Most notably for humans, the North American samples rated humans as much higher on experience than either of the Fijian groups. Both Fijian samples rated humans higher on the two agency factors and lower on the experience factor. The North American samples rated God high on agency and very low on experience, where the Fijian groups rated their gods as high on agency, but more similar to humans on experience. The experience‐basic factor was given neutral ratings by both the Hindus and Muslims—suggesting these groups see God as similar to humans on this dimension. The iTaukei Christians rated God as being higher on the experience factor than humans. The Kalou‐vu were given lower ratings than humans on all factors. Together, this suggests that the iTaukei in our sample did differentiate between different mental factors for different types of agents, despite normative prohibitions against talking about mental states.

### 7.2 Reward and punishment

**Figure 2:**
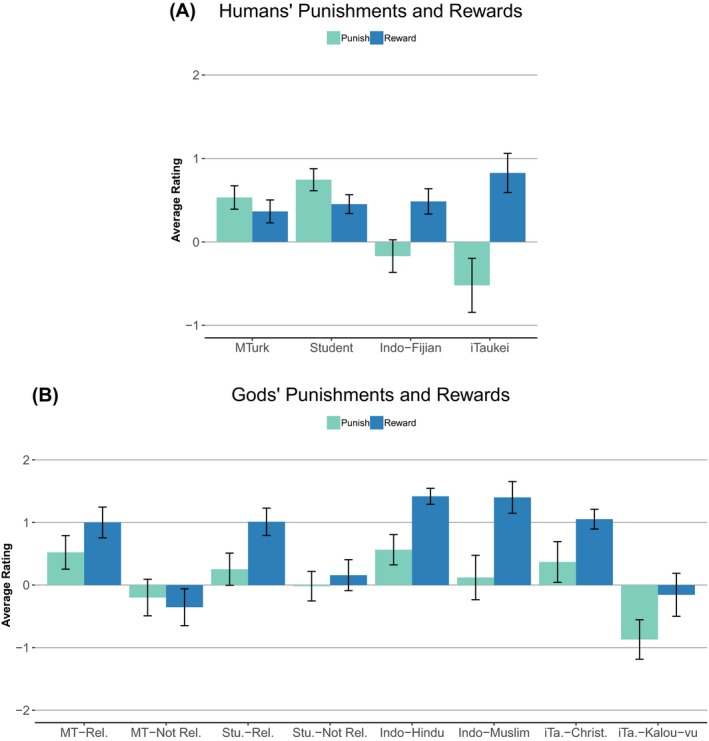
Comparison of how punishing or rewarding tendencies of humans (A) and gods (B) are across samples. Error bars are 95% confidence intervals.

There was substantial variation in the tendency to reward and punish in both humans and gods’ minds across the samples (Fig. 2). The Fijian samples rated humans as less likely to punish bad behavior than the North American samples, and the iTaukei participants rated humans as more likely to reward good behavior than North Americans. All groups, other than the non‐religious North Americans, rated God as more rewarding than punishing. But the Hindus and Muslims rated their gods as more rewarding than the other groups.

We ran a set of regression models to see how the factors ratings of mental states differed in predicting reward and punishment for each group. Since the North American samples and the Fijian samples divide minds up into factors differently, it is difficult to compare between these cultures directly without compromising the factor structure. We explain how we dealt with this for each comparison.

We assessed group differences in the impact of agency and experience factors of human minds on ratings of reward and punishment using the two‐factor agency/experience structure found in the North American samples (see Table 4). This was chosen over the three‐factor model because the agency‐self and agency‐other factors are highly correlated in the North American samples (MTurk: *r *=* *.73, 95% CI: 0.65–0.79; Student: *r *=* *.53, 95% CI: 0.42–0.66). Therefore, separating them for the North American samples would risk excessive collinearity. While these factors do separate for Fijians, they are correlated in the Indo‐Fijians, (*r *=* *.34, 95% CI: 0.21–0.44), but not the iTaukei (*r *=* *0.06, 95% CI: −0.17 to 0.20). This suggests that these factors are even more discrete for the iTaukei sample than the Indo‐Fijian one. Interactions between groups and factors were included to assess group differences in these relationships.

**Table 4:** Agency and experience ratings of humans predicting how much they punish and how much they reward

	Punish—Humans		Reward—Humans	
	β (SE)	95% CI	β (SE)	95% CI
Intercept	0.28 (0.22)	[−0.20, 0.78]	−0.64 (0.25)*	[−1.14, −0.15]
Agency	0.03 (0.07)	[−0.10, 0.16]	0.45 (0.08)***	[0.34, 0.59]
Experience	0.32 (0.08)***	[0.17, 0.46]	0.05 (0.09)	[−0.10, 0.20]
Student	0.15 (0.12)	[−0.06, 0.35]	0.12 (0.14)	[−0.10, 0.35]
Indo‐Fijian	−0.12 (0.11)	[−0.33, 0.09]	0.13 (0.13)	[−0.10, 0.37]
iTaukei	−0.02 (0.20)	[−0.50, 0.42]	0.47 (0.22)*	[0.02, 0.99]
A*Student	0.01 (0.10)	[−0.18, 0.19]	−0.20 (0.11)†	[−0.39, 0.001]
A*Indo	−0.12 (0.09)	[−0.31, 0.07]	−0.15 (0.10)	[−0.35, 0.05]
A*iTau.	−0.14 (0.14)	[−0.49, 0.20]	−0.19 (0.16)	[−0.66, 0.19]
E*Student	0.15 (0.12)	[−0.05, 0.37]	0.16 (0.13)	[−0.09, 0.40]
E*Indo	0.27 (0.10)**	[0.07, 0.47]	−0.24 (0.12)*	[−0.45, −0.03]
E*iTau.	0.48 (0.17)**	[0.02, 0.89]	−0.20 (0.20)	[−0.61, 0.18]

Note

American MTurk sample was used as the reference category. ^†^ *p* < .10, *p < .05, **p < .01, ***p < .001. Additional controls variables not shown: age, gender, and years of formal education.

If agency and experience predict reward and punishment in the same way across groups, then controlling for agency and experience factors should eliminate group‐level differences in these variables. We found some evidence against this with the iTaukei participants who rated reward higher than the MTurk sample (β = 0.47). This suggests something additional to agency and experience is accounting for why the iTaukei sample rates humans as tending to reward others for good behavior. We also found evidence that these factors related to reward and punishment differently in our Indo‐Fijian sample than in our North Americans. The Indo‐Fijians showed a stronger relationship between the experience factor and punishment (β = 0.27) and a weaker relationship between the experience factor and reward (β = −0.24) than the MTurk sample.

If the iTaukei were not using mental states to predict reward and punishment behavior, as the Strong Opacity prediction would suggest, there should be no relationship between agency or experience and these variables. This would be seen in the interaction terms, which should be in opposition to the effects for the MTurk sample (the main effects of agency and experience). This is not the case. iTaukei participants do show an interaction for the experience factor predicting punishment, but this interaction is positive not negative (β = 0.48), suggesting the relationship between the experience factor and punishment is stronger for the iTaukei sample than for the student sample. There was an interaction effect for iTaukei participants using the agency factor to predict reward (β = −0.20, *n.s*.) that does counter the main effect of the agency factor. This effect was both non‐significant and of a similar magnitude to the interaction found in the Indo‐Fijian sample, opening the possibility that it might be caused by some other cultural trait than Opacity norms. We analysed the iTaukei sample alone (Table 5; see Appendix S1 for other groups) and found that the experience factor significantly predicted punishment (β = 0.80). This supports the idea that the iTaukei sample does indeed relate internal mental states to the tendency to punish.

**Table 5:** Two agency factors and experience predicting reward and punishment in iTaukei sample

	Punishment		Reward	
	β (*SE*)	[95% CI]	β (*SE*)	[95% CI]
Intercept	0.55 (0.72)	[−0.80, 2.15]	0.87 (0.74)	[−0.50, 2.13]
Agency—other	−0.07 (0.12)	[−0.29, 0.17]	−0.005 (0.12)	[−0.25, 0.27]
Agency—self	−0.08 (0.14)	[−0.34, 0.20]	0.27 (0.15)	[−0.11, 0.60]
Experience	0.83 (0.20)***	[0.30, 1.24]	−0.23 (0.20)	[−0.61,0.13]

Note

***p < .001. Additional controls variables not shown: age, gender, and years of formal education.

We split up the analysis of gods’ minds to facilitate meaningful comparisons across groups. First, we compared the iTaukei Christians, non‐religious North Americans, and Christian North Americans (this analysis excluded the non‐Christian religious participants).^2^ This allowed us to directly compare how Christians from different cultural backgrounds think about the mind of god as well as look at a non‐religious sample (see Table 6). To do this, we again used the agency and experience factors from the North American sample, with Christians from MTurk as the reference category. This combined the two experience factors found for God's mind among the Fijians. These two experience factors show a medium correlation in the iTaukei sample (*r *=* *.48, 95% CI: 0.29–0.64). Second, we compared Hindu and Muslim Indo‐Fijians to see if people from a similar cultural background, but different religious groups, would use these factors differently to assess God's mind. Finally, we assessed how these factors related to reward and punishment variables in iTaukei participants’ Kalou‐vu beliefs.

**Table 6:** Agency and experience ratings of God predicting the extent to which God punishes and rewards

	Punishment		Reward	
	β (*SE*)		β (*SE*)	
*North American Christians and non‐religious, and iTaukei Christians, n = 376*				
Intercept	0.15 (0.34)	[−0.54, 0.83]	−0.05 (0.26)	[−0.55, 0.45]
Agency	0.44 (0.12)***	[0.15, 0.64]	0.85 (0.09)***	[0.72, 1.05]
Experience	0.31 (0.11)**	[0.10, 0.53]	0.12 (0.09)	[−0.05, 0.30]
Mtu. Not Rel.	0.23 (0.14)	[−0.07, 0.50]	−0.02 (0.11)	[−0.29, 0.23]
Stu. Christ.	−0.34 (0.20)†	[−0.76, 0.06]	0.01 (0.15)	[−0.25, 0.26]
Stu. Not Rel	−0.04 (0.16)	[−0.34, 0.27]	0.01 (0.12)	[−0.21, 0.24]
iTau. Christ.	−1.06 (0.26)***	[−1.57, −0.52]	0.30 (0.19)	[−0.03, 0.63]
A*Mtu. Not Rel.	−0.001 (0.14)	[−0.30, 0.31]	−0.17 (0.11)	[−0.44, 0.04]
A*Stu. Christ.	−0.09 (0.21)	[−0.53, 0.36]	−0.17 (0.16)	[−0.53, 0.07]
A*Stu. Not Rel.	−0.06 (0.14)	[−0.34, 0.27]	−0.07 (0.11)	[−0.29, 0.11]
A*iTau. Christ	0.02 (0.36)	[−0.77, 0.87]	−0.44 (0.27)†	[−0.84, −0.02]
E*Mtu. Not Rel.	0.06 (0.15)	[−0.23, 0.37]	0.02 (0.12)	[−0.22, 0.28]
E*Stu. Christ.	−0.36 (0.18)†	[−0.76, 0.06]	−0.33 (0.14)*	[−0.66, −0.05]
E*Stu. Not Rel.	−0.07 (0.16)	[−0.36, 0.23]	−0.21 (0.11)†	[−0.43, 0.01]
E*iTau. Christ	0.69 (0.19)***	[0.27, 1.02]	−0.15 (0.15)	[−0.39, 0.08]
*Indo*‐*Fijian Hindus and Muslims, n = 214*				
Intercept	0.59 (0.29)*	[0.05, 1.11]	−0.29 (0.16)†	[−0.64, 0.03]
Agency	0.06 (0.17)	[−0.31, 0.46]	0.43 (0.10)***	[0.14, 0.69]
Exp. Motive	0.46 (0.10)***	[0.26, 0.62]	0.06 (0.06)	[−0.05, 0.17]
Exp. Basic	−0.35 (0.09)***	[−0.51, −0.18]	0.02 (0.05)	[−0.05, 0.08]
Muslim	−0.17 (0.19)	[−0.52, 0.23]	−0.17 (0.11)	[−0.47, 0.13]
A*Muslim	−0.35 (0.26)	[−0.94, 0.22]	0.61 (0.25)***	[0.15, 1.04]
ES*Muslim	−0.03 (0.18)	[−0.42, 0.38]	−0.15 (0.10)	[−0.32, 0.02]
EB*Muslim	−0.02 (0.13)	[−0.32, 0.29]	0.01 (0.08)	[−0.10, 0.12]
*iTaukei Kalou‐vu, n = 77*				
Intercept	−0.25 (0.69)	[−1.65, 1.14]	−0.07 (0.62)	[−1.49, 1.30]
Agency	0.02 (0.23)	[−0.45, 0.46]	0.69 (0.21)**	[0.27, 1.08]
Exp. Social	0.57 (0.25)*	[0.02, 1.06]	−0.11 (0.23)	[−0.54, 0.36]
Exp. Basic	−0.12 (0.24)	[−0.60, 0.41]	0.62 (0.21)**	[0.12, 1.08]

Note

Christians from MTurk were reference category in first analysis and Hindus were reference category in second analysis. ^†^ *p *< .10, *p < .05, **p < .01, ***p < .001. Additional controls variables not shown: age, gender, and years of formal education.

Christian and non‐religious participants across iTaukei and North American samples related factors of mental states differently to God's tendency to punish than they did for humans. Unlike the predictions for humans, ratings of God's agency predicts belief in God's tendency to punish across all groups. The relationship between experience and punishment is maintained in all groups but the Christian students. We found a marginally lower relationship between God's experience and tendency to punish for Christians students than the Christians from MTurk (β = −0.36). The relationship between experience and God's tendency to punish was stronger in the iTaukei sample than for Christians from MTurk (β = 0.69). Again, we can conclude that the iTaukei sample is using the experience factor to predict punishment tendencies despite Opacity norms. The iTaukei and both Christian and non‐religious students show a weaker relationship between agency and God's tendency to reward than the MTurk sample.

We next looked at the ratings for non‐Christian supernatural agents (Hindus, Muslims, and separately iTaukei ratings of the Kalou‐vu). We found that experience predicted gods’ tendencies to punish, but this was positive for the experience‐social factor (β = 0.46) and negative for the experience‐basic factor (β = −0.35). Unlike the previous analysis, the agency factor did not predict the gods’ tendencies to punish in any of these groups. The experience‐basic factor predicted iTaukei participants’ reward ratings for the Kalou‐vu (β = 0.62), while only the agency factor predicted Indo‐Fijians’ divine reward ratings (β = 0.43). Muslim participants showed a stronger relationship between the agency factor and reward than Hindu participants (β = 0.61). The differing use of the two experience factors further supports them as independent factors that are not easily reducible to a single dimension. These findings taken together suggest that mentalistic capacities that describe belief in God in research from North America may not be generalizable to other societies, even when those societies are also part of the same wider religious tradition.

### 7.3 Predicting Gods’ body and mind

Finally, to assess the relationship between our mental factors, physicality, and gods’ supernatural mindreading abilities, we asked participants a question about whether or not God has a physical body and a question about whether or not God can know your thoughts. There was substantial variation in answers to whether or not God has a body, but much less in the mind reading question (Table 7). Only the non‐religious North Americans and the iTaukei ratings of the Kalou‐vu showed any substantial disagreement.

**Table 7:** Percentage of samples reporting belief that God has a physical body and can know the participant's thoughts

	% “Yes” God Has a Physical Body	% “Yes” God Can Know Your Thoughts
Students—Religious	17.6	93.4
Students—Not Relig.	10.1	48.6
MTurk—Religious	18.9	91.2
MTurk—Not Relig.	7.4	40.0
Indo‐Hindu	54.0	95.0
Indo‐Muslim	2.8	100.0
iTaukei Christian	45.9	100.0
iTaukei Kalou‐vu	46.7	39.0

We used ratings of agency and experience, as factored by each sample, to predict the likelihood of believing God has a physical body in samples with enough variation to make meaningful predictions (Table 8). The high level of “yes” answers to the question about gods’ mindreading abilities prevents us from predicting these answers in any sample but the iTaukei Kalou‐vu and the non‐religious North Americans.

**Table 8:** Binary logistic regression table predicting odds of reporting belief that God has a body and can know one's thoughts

	β (*SE*)	OR	[95% CI]
God has a physical body			
*North American*—*Religious*			
Agency	0.02 (0.39)	1.20	[0.47, 2.21]
Experience	0.08 (0.36)	1.08	[0.54, 2.19]
MTurk Rel.	0.52 (0.64)	1.68	[0.47, 5.99]
Agen*Mt. Rel.	0.12 (0.56)	1.12	[0.37, 3.39]
Exp*Mt. Rel.	0.51 (0.54)	1.66	[0.58, 4.78]
*Hindu*			
Agency	0.49 (0.43)	1.64	[0.71, 3.81]
Exp. Social	−1.34 (0.32)***	0.26	[0.14, 0.50]
Exp. Basic	−0.06 (0.26)	0.94	[0.57, 1.56]
*iTaukei Christ*.			
Agency	−1.32 (0.91)	0.27	[0.05, 1.59]
Exp. Social	−0.01 (0.56)	0.99	[0.34, 2.99]
Exp. Basic	−0.48 (0.49)	0.62	[0.23, 1.63]
*iTaukei Kalou‐vu*			
Agency	0.26 (0.43)	1.29	[0.56, 2.99]
Exp. Social	0.78 (0.57)	2.17	[0.71, 6.62]
Exp. Basic	0.44 (0.52)	1.55	[0.56, 4.26]
God knows your mind			
*North American*—*non‐Religious*			
Agency	2.11 (0.40)***	8.22	[3.73, 18.11]
Experience	−0.73 (0.40)†	0.48	[0.22, 1.05]
MTurk Not Rel.	0.62 (0.69)	0.48	[0.49, 7.16]
Agen*Mt. Not Rel.	0.51 (0.71)	1.66	[0.41, 6.73]
Exp*Mt. Not Rel.	0.09 (0.63)	1.09	[0.32, 3.72]
*iTaukei Kalou‐vu*			
Agency	0.14 (0.42)	1.51	[0.98, 2.33]
Exp. Social	−0.001 (0.55)	0.79	[0.44, 1.39]
Exp. Basic	0.64 (0.51)	1.07	[0.71, 1.62]

Note

Groups without enough variance to analyze have been excluded from analysis. †*p *< .10, ***p < .001. Additional controls variables not shown: age, gender, and years of formal education.

Among the Hindu participants, viewing God as having social experience negatively predicted thinking God had a body. This is the opposite of what we predicted based on previous research. It suggests that experience is not always positively related to this type of physicality/emotionality. Rather, our Hindu participants who rated God as higher in capacities like anger, pain, and pleasure are less likely to think of God as having a physical body. This was the only effect found predicting answers to the question about gods’ physical bodies. The non‐religious North Americans showed the expected highly agentic mentalistic pattern: When God is rated as more agentic and less experiential, God is also rated as more likely to know what is going on in a person's mind. This may simply reflect level of belief; people who believe a bit are more likely to suggest that God can know their thoughts and more likely to endorse the culturally sanctioned belief that God is all agency and little experience. No significant effects were found for the Kalou‐vu.

## 8 Discussion

Despite the cultural variance in our sample, and Opacity of Mind norms in one of our samples, our factor analysis broadly corresponds to the agency and experience dimensions found in Gray et al. (2007), pointing to some degree of consistency across these cultural groups. Still, there are some important differences between our samples that cannot be reduced to the original two factors found in Gray et al. (2007)'s research. In the Fijian sample, we found three factors, rather than two, for both gods and humans minds. Though these three factors do not completely rewrite the dimensions of mind perception found by Gray et al. (2007), they do split them into additional pieces. At the same time, the factor structures we found cannot be reduced to the agency and experience dimensions without producing non‐meaningful results.

The splitting of human agency into agency‐other and agency‐self, as show in the Fijian samples, may reflect a relational understanding of mind—that mind exists in the space between people rather than just within an individual (see ojalehto et al., 2017b). This emphasis on a relational, sociocentric model of self is important throughout the Pacific (Airni, Anae, & Mila‐Schaaf, 2010; Anae, 2010; Brison, 2001; Poltorak, 2007) and may be filtering into the processes of how people conceive of minds that we measure here. Given that we do not find support for the Strong Opacity prediction of no relationship between mind perception and behavioral expectations, it is likely that these sociocentric models inform person perception for both our iTaukei and Indo participants. This idea that a relational cultural environment could shape the perception of separation between other and self‐focused mental capacities is a promising area for further cross‐cultural research.

Other research has found three factors in a North American sample (Weisman et al., 2017). This three‐factor break down splits mental factors in to body, heart, and mind—corresponding to physiological, socio‐emotional, and mentalistic dimensions of the mind. Weisman et al.'s (2017) study used more items than Gray et al. (2007) or our study. These additional items may account for the difference in factors. Weisman et al.'s (2017) factors correspond more closely to the factors we found in the Fijian sample rating characteristics of human minds—with heart being similar to agency‐other and mind being similar to agency‐self. Still, this cannot account for the differences between how North Americans and Fijians conceive of human minds in our study, nor why Fijians would conceive of the mind of gods differently from human minds.

Fijians’ ratings of gods’ minds factored differently than their ratings of human minds, dividing experience rather than agency into social and basic factors. Based on this limited set of minds, Fijians do not appear to use a single set of dimensions for perceiving minds, but see mental capacities as clustering together differently depending on the type of mind they are assessing. Gods do not simply have a bigger better version of the human mind; they have a different *type* of mind. Moreover, Fijians did not rate gods as all agency nor humans as higher than gods in experience like the North American samples did. This cannot be solely accounted for based on differences between religions. The iTaukei Christians give God's mind the highest ratings of experience of any of the religious groups, and more experience than humans. This can be directly contrasted with findings from our religious North American participants and previous work on the conception of God's mind in Western samples. Conceptions of gods’ minds are not invariant across cultures, even within a single religious tradition. This throws into question many of the generalizations about religion, even Christianity specifically, that have been generated using research on only North American participants.

The iTaukei participants’ ratings of Kalou‐vu minds show that they think of these minds as lower on all factors than human minds. Though these are the spirits of ancestors and not gods in the strictest sense, it does suggest that religiously generated supernatural minds may not always be represented as superhuman (cf. Haslam et al., 2008). It is worth noting that we cannot conclude from these data exactly how our iTaukei sample conceives of the dimensionality of minds for either supernatural agents or humans, as our sample was not large enough to run a factor analysis on iTaukei participants alone. All we can say is that the factor structure does not change when the iTaukei are added to the Indo‐Fijian sample.

Unlike the Fijians, the North American samples’ ratings produced the same two dimensions of mind for God's mind and human's minds. It has long been claimed that God, particularly the Christian one, is an anthropomorphic concept (Feuerbach, 1957; Guthrie, 1993, 1996; Hume, 1779/1981), and it could be argued that the longer history of Christianity in the North American samples has created a more anthropomorphic concept of God. This does not clearly follow from our data. Though the factors are the same for both God and humans in the North American samples, these samples rate God as much higher in agency and much lower in experience than humans, suggesting that, despite the similar dimensionality of mind perception at play, God's mind is seen as having different strengths and qualities than human minds. This paired with Fijians’ tendency to rate their gods’ minds as higher than, or similar to, human minds on all factors suggests that Fijians see their gods’ minds as more superhuman than North Americans do, and North Americans see God's mind as a type of highly agentic mind rather than a bigger better human mind. Instead of reflecting an anthropomorphic tendency, the similarly in factor structure across minds might reflect a greater desire for consistency in the North American sample than the Fijian one (see Markus & Kitayama, 1991; Suh, 2002). Some work suggests that North Americans' implicit conceptions of God are more anthropomorphic than their explicit ones (see Heiphetz et al., 2015 for a review), and we only look at explicit conceptions here.

We did not find any clear evidence that Opacity of Mind norms reduce the iTaukei Fijian participants’ willingness to attribute mental states to either humans or God. In fact, the iTaukei sample was more willing to attribute agency‐related mental capacities to humans than the Indo‐Fijian or North American student sample. Experience capacities for humans—like anger, fear, and desire—garnered much more neutral ratings on average for both Fijian groups. If there was any reluctance in allocating mental states to humans by the iTaukei it was in these sorts of capacities, but the similar ratings in the Indo population makes it difficult to clearly relate this to Opacity norms. Further, factor allocations for God's mind reliably predicted ratings of punishment in the iTaukei sample.

If the Light Opacity prediction is true—that Opacity norms are about politeness and invasion of privacy—then one might expect people to be reluctant to answer questions about the mental states of specific people rather than humans as a category. This willingness to attribute mental states to humans in general may simply reflect a normative understanding of people for our iTaukei participants. They may rely upon normative understanding in place of reasoning about the specific mental states of particular individuals in particular contexts. This may further reflect a general perception that others have active mental lives along with a deep skepticism that these mental states—contained within the “opaque” container of the mind—can ever really be known by another person. Together this suggests that these restrictions may dissuade iTaukei Fijians from discussing the internal mental states of specific others while not affecting how much they relate mental states to humans or God in general. This also suggests some type of universality to conceptions of minds. Even when they are normatively restricted, they are still reliably developing, but culture does seem to impact how these capacities are seen to relate to one another.

It is worth noting that our methods differed from the original Gray et al. (2007) paper, both in how the ratings were taken (we used scales rather than binary comparisons) and the number of agents (they used substantially more agents than we did). It is possible that the differences in our findings are due to the agents we picked and would be less obvious had we averaged across a wider range of agents. This proposition, though plausible, still suggests that the factor structure of minds would differ based on the type of agents that are being evaluated even if on average they can be summed into a two‐factor division. Given the difference in our and others' (see Weisman et al., 2017) work from the original findings, more research of this type is clearly needed. Specifically, we need more cross‐cultural work before we can pinpoint a particular underlying dimensionality of mind perception, while more culturally nuanced work is needed in order to understand how we conceive of minds in various socio‐cultural environments.

When we used the agency and experience factor structures to predict beliefs about the reward and punishment tendencies of humans and supernatural agents, we again found that both cultural and religious backgrounds matter. Though our iTaukei participants and many of our religious North Americans share the belief system of Christianity, we found that this shared religious background is insufficient to create a shared perception of how agency and experience predict God's reward and punishment tendencies. The differences between the Muslim and Hindu Indo‐Fijians further suggest that different religions within an ethnic group can impact how individuals perceive divine minds and their relation to morally relevant tendencies.

Moving from gods’ minds to gods’ bodies, only our Hindu sample contains any significant relationship between ratings of minds and beliefs about God's physical body. This relationship is counter to what previous research would, and what we did, predict. Those who rate God as being higher in agency and lower in one of the experience factors are more likely to say God has a body. The belief that God has a body is negatively related to ratings of emotion and sensory experience. Though this cannot speak directly to previous work, which shows that more emphasis of human bodies relates to lower ratings of agency and higher ratings of experience (Gray et al., 2011), we can say this relationship does not appear in reverse. Higher ratings of experience do not relate to a greater probability of Gods having a body in our samples. It is unclear what the 17%–18% of religious North Americans and the 46% of iTaukei Christians who thought God had a body were thinking about, but it is possible they were thinking about Jesus rather than God. Regardless, we found no effects in these groups.

## 9 Conclusion

The many decades of systematic observation and experimentation in Western samples paints a fairly coherent picture of how participants conceive of others’ minds. However, when we expand our research to other societies, we come across new and interesting ways that humans can conceive of different types of minds. This paper presents only a small sliver of the cultural diversity seen around the world, and only a small part of the complex sets of characteristics and abilities that make up mind perception, and yet we find quite a large amount of diversity across all of our analyses. Whether we looked at how concepts of minds are structured or how these structures are used to predict other variables, cultural differences mattered. Much more cross‐cultural work is needed before we can confidently make any universal claims about how humans conceive of minds, and more attention needs to be paid to specific historical differences in cultural context. For example, the long philosophical tradition focused on reason may have made European‐origin cultures more likely to denigrate emotional experience and elevate reason and intellect in a way not seen in other parts of the world (e.g., Nisbett, Peng, Choi, & Norenzayan, 2001). The conception of God as all agency and no experience in the West may reflect this ideal.

## Supporting information


**Appendix S1.** The minds of god(s) and humans: Supplemental.

## Notes

1 When a two‐factor solution is forced, plans/goals, thinking, and self‐control do not load onto either factor.

2 We have removed the non‐Christian religious participants from this sample so as to more accurately compare our three Christian groups. Including all religious participants does not affect the results in any meaningful way. See Appendix S1 for this analysis.
