# Supplementary material for: The Minds of God(s) and Humans: Differences in Mind Perception in Fiji and North America
Source: Cogn Sci. 2019 Jan 7;43(1):e12703. doi: 10.1111/cogs.12703 (PMC6590269; doi:10.1111/cogs.12703)
Supplement: Supplementary file 1 — Appendix S1. The minds of god(s) and humans: Supplemental. [file COGS-43-na-s001.docx]

# The Minds of God(s) and Humans: Supplemental

Data for this project can be found at: https://osf.io/dyjpq/

## Human and God’s minds questionnaires (used in all samples)

| Humans |
| --- |
| 1. How good are most people at understanding how others are feeling? |
| 1. How good are most people at thinking? |
| 1. How good are most people at exercising self-control? |
| 1. How capable are most people at having experiences and being aware of things? |
| 1. How capable are most people at telling right from wrong? |
| 1. How good are most people at remembering things? |
| 1. How good are most people at making plans and working towards a goal? |
| 1. How good are most people at experience fear? |
| 1. How good are most people at desire material possession? |
| 1. How good are most people at experience embarrassment? |
| 1. How much do most people feel hunger? |
| 1. How much do most people experience pride? |
| 1. How much do most people desire attention from others? |
| 1. How much do most people hope or wish for things? |
| 1. How much do most people feel pain? |
| 1. How much do most people experience anger? |
| 1. How much do most people experience joy? |
| 1. How much do most people feel pleasure?   Reward and Punish   1. How much to most people reward other people for their good behavior? 2. How much to most people punish other people for their bad behavior? |

| God |
| --- |
| 1. Compared to most people, how good is God at understanding how others are feeling? |
| 1. Compared to most people, how good is God at thinking? |
| 1. Compared to most people, how good is God at exercising self-control? |
| 1. Compared to most people, how capable is God at having experiences and being aware of things? |
| 1. Compared to most people, how capable is God at telling right from wrong? |
| 1. Compared to most people, how good is God at remembering things? |
| 1. Compared to most people, how good is God at making plans and working towards a goal? |
| 1. Compared to most people, how much does God experience fear? |
| 1. Compared to most people, how much does God desire material possession? |
| 1. Compared to most people, how much does God experience embarrassment? |
| 1. Compared to most people, how much does God feel hunger? |
| 1. Compared to most people, how much does God experience pride? |
| 1. Compared to most people, how much does God desire attention from others? |
| 1. Compared to most people, how much does God hope or wish for things? |
| 1. Compared to most people, how much does God feel pain? |
| 1. Compared to most people, how much does God experience anger? |
| 1. Compared to most people, how much does God experience joy? |
| 1. Compared to most people, how much does God feel pleasure?   Reward and Punish   1. Compared to most people, how much does God reward people for their good behavior? 2. Compared to most people, how much does God punish people for their bad behavior? |

## Factor analysis of Human minds using only Indo Fijians

| **Indo- Human** |  |  |  | |
| --- | --- | --- | --- | --- |
| **Item** | **Experience** | **Agency-other** | **Agency-self** | |
| Desire material | 0.62 | -0.10 | 0.11 | |
| Fear | 0.59 | -0.05 | -0.23 | |
| Hunger | 0.58 | 0.02 | 0.14 | |
| Desire attention | 0.58 | -0.11 | 0 | |
| Pain | 0.55 | -0.04 | -0.27 | |
| Anger | 0.52 | 0.07 | -0.14 | |
| Pride | 0.46 | -0.12 | 0.33 | |
| Embarrassed | 0.32 | 0.35 | -0.25 | |
| Hope/wish | 0.23 | 0.08 | -0.55 | |
| Aware | -0.04 | 0.81 | -0.06 | |
| Tell right/wrong | -0.02 | 0.68 | 0.03 | |
| Others feelings | -0.08 | 0.59 | 0.23 | |
| Remembering | -0.25 | 0.43 | 0.01 | |
| Pleasure | 0.22 | 0.33 | -0.04 | |
| Joy | 0.17 | 0.28 | -0.15 | |
| Thinking | -0.12 | 0.19 | 0.46 | |
| Plans/goals | 0.26 | 0.25 | 0.42 | |
| Self control | -0.03 | 0.28 | 0.41 | |
| **Factor correlations & variance** | | | |  |
|  | **E** | **A-O** | **A-S** | |
| Experience | (0.90) |  |  | |
| Agency-other | -0.24 | (0.90) |  | |
| Agency-self | -0.28 | 0.17 | (0.82) | |
| Prop. Variance | 0.15 | 0.13 | 0.08 | |

Table 1: Exploratory Factor analysis of human question using only Indo-Fijian subjects. Loadings of 0.60 or higher are highlighted in dark gray; loadings of 0.30 or higher are highlighted in medium gray; and cross loadings of 0.30 or higher are highlighted in light grey.

## Factor analysis of God’s mind with alternative breakdowns of the Fijian samples.

| **Hindu only** |  |  |  |
| --- | --- | --- | --- |
| **Item** | **Exp. Social** | **Exp. Basic** | **Agen.** |
| Hope/wish | 0.71 | -0.03 | 0.00 |
| Pride | 0.68 | 0.14 | 0.05 |
| Embarrassed | 0.59 | 0.04 | -0.10 |
| Anger | 0.58 | 0.04 | 0.03 |
| Pain | 0.39 | -0.27 | -0.05 |
| Pleasure | 0.37 | -0.20 | 0.19 |
| Desire Attention | 0.34 | -0.01 | 0.29 |
| Desire Material | -0.01 | 0.83 | 0.03 |
| Fear | -0.04 | 0.71 | 0.04 |
| Hunger | 0.22 | 0.61 | -0.16 |
| Aware | 0.03 | 0.05 | 0.79 |
| Others feelings | -0.15 | -0.04 | 0.70 |
| Remembering | -0.09 | 0.12 | 0.70 |
| Know right/wrong | 0.11 | -0.16 | 0.65 |
| Thinking | 0.17 | -0.05 | 0.52 |
| Joy | 0.09 | -0.04 | 0.51 |
| Plans/goals | 0.09 | -0.06 | 0.40 |
| Self control | 0.33 | 0.13 | 0.32 |
| **Factor Corr.** |  |  |  |
|  |  |  |  |
| Exp. Social | (0.90) |  |  |
| Exp. Basic | 0.19 | (0.90) |  |
| Agency | 0.16 | -0.17 | (0.92) |
|  |  |  |  |

Table 2 Exploratory Factor analysis of God question using only Hindu subjects. Loadings of 0.60 or higher are highlighted in dark gray; loadings of 0.30 or higher are highlighted in medium gray; and cross loadings of 0.30 or higher are highlighted in light grey.

| **Abrahamic God (iTaukei and Muslims)** | | | |
| --- | --- | --- | --- |
| **Items** | **Exp. Social** | **Exp. Basic** | **Agency** |
| Hope/wish | 0.73 | -0.05 | 0.09 |
| Embarrassed | 0.43 | 0.09 | -0.01 |
| Pleasure | 0.38 | -0.06 | 0.28 |
| Pain | 0.30 | 0.11 | 0.27 |
| Anger | 0.20 | 0.20 | 0.07 |
| Desire material | -0.06 | 1.00 | 0.01 |
| Fear | 0.28 | 0.55 | 0.02 |
| Hunger | 0.41 | 0.43 | -0.19 |
| Thinking | -0.16 | 0.14 | 0.73 |
| Others feelings | -0.03 | -0.12 | 0.70 |
| Aware | 0.03 | 0.00 | 0.65 |
| Plans/goals | -0.05 | -0.03 | 0.59 |
| Desire attention | 0.21 | 0.07 | 0.54 |
| Remember | 0.13 | -0.07 | 0.51 |
| Joy | 0.13 | -0.07 | 0.51 |
| Tell right/wrong | 0.17 | 0.01 | 0.46 |
| Self control | 0.17 | -0.02 | 0.29 |
| Pride | 0.01 | 0.21 | 0.27 |
| **Factor Corr.** |  |  |  |
|  |  |  |  |
| Exp. 1 | (0.84) |  |  |
| Exp. 2 | 0.19 | (1.00) |  |
| Agency | 0.14 | -0.05 | (0.92) |
|  |  |  |  |

Table 3: Exploratory Factor analysis of God question using Muslim and indigenous Fijian Christian subjects. Loadings of 0.60 or higher are highlighted in dark gray; loadings of 0.30 or higher are highlighted in medium gray; and cross loadings of 0.30 or higher are highlighted in light grey.

## Factor breakdown including reward and punishment questions

When reward and punishment are included in the factor analyses (will all other items) they load on factors in the following way:

| **North America - Human** | | |  | **Fiji - Human** | | | |
| --- | --- | --- | --- | --- | --- | --- | --- |
| **Item** | **Exp.** | **Agency** |  | **Item** | **Exp.** | **Agency-self** | **Agency-other** |
| Reward | 0.14 | 0.44 |  | Reward | -0.15 | 0.19 | 0.20 |
| Punishment | 0.44 | 0.03 |  | Punishment | 0.62 | -0.02 | -0.01 |

| **North America - God** | | |  | **Fiji - God** | | | |
| --- | --- | --- | --- | --- | --- | --- | --- |
| **Item** | **Exp.** | **Agency** |  | **Item** | **Exp.-Social** | **Exp.-Basic** | **Agency** |
| Reward | 0.01 | 0.82 |  | Reward | 0.02 | 0.02 | 0.59 |
| Punishment | 0.17 | 0.55 |  | Punishment | 0.52 | -0.20 | -0.04 |

## Group differences in factors and reward and punishment questions

|  | **Agency Other** | **Agency Self** | **Experience** | **Punish** | **Reward** |
| --- | --- | --- | --- | --- | --- |
|  | **β (SE)**  **[95% CI]** | **β (SE)**  **[95% CI]** | **β (SE)**  **[95% CI]** | **β (SE)**  **[95% CI]** | **β (SE)**  **[95% CI]** |
| ***Human*** |  |  |  |  |  |
| Intercept | -0.71 (0.26)** | 0.53 (0.26)* | 0.17 (0.21) | 0.23 (0.24) | -0.55, (0.26)* |
|  | [-1.30, -0.18] | [0.02, 1.05] | [-0.28, 0.59] | [-0.56, 0.59] | [-1.35, -0.12] |
| Student | 0.11 (0.12) | 0.02 (0.12) | 0.19 (0.10)† | 0.33 (0.12)** | 0.24 (0.12)* |
|  | [-0.12, 0,35] | [-0.20, 0.59] | [-0.01, 0.40] | [0.14, 0.53] | [0.01, 0.47] |
| Indo-Fijian | 0.18 (0.12) | -0.11 (0.12) | -1.03 (0.10)*** | -0.55 (0.11)*** | 0.24 (0.12)* |
|  | [-0.06, 0.41] | [-0.37, 0.15] | [-1.26, -0.81] | [-0.76, -0.32] | [-0.003, 0.47] |
| iTaukei | 0.53 (0.17)** | 0.27 (0.17) | -1.23 (0.14)*** | -0.82 (0.16)*** | 0.71 (0.17)*** |
|  | [0.13, 0.90] | [-0.05, 0.59] | [-1.48, -0.96] | [-1.15, -0.45] | [0.35, 1.05] |
| ***God*** | **Agency** | **Exper. Social** | **Exper. Basic** |  |  |
| Intercept | 0.68 (0.22)** | 0.03 (0.24) | -0.68 (0.23) | 0.56 (0.26)* | 0.17 (0.22) |
|  | [0.26, 1.09] | [-0.39, 0.45] | [-1.13, -0.23] | [0.08, 1.05] | [-0.27, 0.61] |
| Mtu. Not Rel. | -1.28, (0.12)*** | -0.47 (0.13)*** | 0.19 (0.13) | -0.51 (0.14)*** | -1.04 (0.12)*** |
|  | [-1.58, -0.99] | [-0.74, -0.20] | [-0.05, 0.44] | [-0.78, -0.23] | [-1.31, -0.73] |
| Stu. Rel. | -0.06 (0.14) | -0.06 (0.14) | -0.28 (0.14)* | -0.32 (0.16)* | -0.03 (0.14) |
|  | [-0.31, 0.19] | [-0.33, 0.21) | [-0.53, -0.03] | [-0.61, 0.02] | [-0.27, 0.25] |
| Stu. Not Rel. | -0.93 (0.13)*** | -0.39 (0.14)** | -0.10 (0.13) | -0.50 (0.15)*** | -0.68 (0.13)*** |
|  | [-1.21, -0.64] | [-0.66, -0.13] | [-0.36, 0.13] | [-0.77, -0.21] | [-0.95, -0.40] |
| Muslim | -0.09 (0.14) | 0.63 (0.15)*** | 0.89 (0.14)*** | -0.35 (0.15)* | 0.29 (0.14)* |
|  | [-0.34, 0.17] | [0.35, 0.90] | [0.57, 1.20] | [-0.69, -0.01] | [-0.002, 0.57] |
| Hindu | 0.03 (0.13) | 0.70 (0.14)*** | 1.01 (0.13)*** | 0.02 (0.14)* | 0.31 (0.12)* |
|  | [-0.17, 0.26] | [0.46, 0.95] | [0.74, 1.28] | [-0.25, 0.30] | [0.09, 0.56] |
| iTa. - Christ. | -0.25 (0.16) | 0.76 (0.16)*** | 1.85 (0.16)*** | -0.14 (0.18) | 0.09 (0.16) |
|  | [-0.45, -0.01] | [0.51, 1.02] | [1.84, 2.41] | [-0.50, 0.23] | [-0.15, 0.36] |
| Note: *p<0.05, **p<0.01, ***p<0.001. Additional controls: Gender, Age and Education. Students were the comparison category for human and Religious students were the comparison for God. Kalou-vu ratings were not included because they were rated by the same sample that rated God. | | | | | |

Table 4: Group difference in factors and reward and punishment questions for humans and God

## Alternative analysis of Reward and Punishment Questions

|  | **Punishment** | | **Reward** | |
| --- | --- | --- | --- | --- |
|  | **β (SE)** | **[95% CI]** | **β (SE)** | **[95% CI]** |
| *Students* |  |  |  |  |
| Intercept | 1.46 (0.72)* | [0.22, 2.79] | -0.93 (0.82) | [-2.59, 0.50] |
| Agency | -0.05 (0.06) | [-0.08, 0.17] | 0.26 (0.07)*** | [0.10, 0.41] |
| Experience | 0.49 (0.87)*** | [0.35, 0.64] | 0.23 (0.09)* | [0.04, 0.40] |
| *M-turk* |  |  |  |  |
| Intercept | 0.49 (0.41) | [-0.28, 1.28] | 0.24 (0.47) | [-1.16, 0.60) |
| Agency | 0.04 (0.06) | [-0.08, 0.18] | 0.47 (0.06)*** | [0.34, 0.59] |
| Experience | 0.32 (0.07)*** | [0.17, 0.46] | 0.06 (0.08) | [-0.08, 0.21] |
| *Indo-Fijian* |  |  |  |  |
| Intercept | -0.09 (0.30) | [-0.72, 056] | -0.62 (0.33)† | [-1.30, 0.04] |
| Agency - other | -0.04 (0.07) | [-0.18, 0.09] | 0.16, 0.08† | [-0.04, 0.35] |
| Agency - self | -0.07 (0.08) | [-0.24, 0.11] | 0.17, 0.08)* | [0.001, 0.33] |
| Experience | 0.55 (0.08)*** | [0.41, 0.68] | -0.19 (0.08)* | [-0.35, 0.03] |
| *Note:* †*p*<0.10, **p*<0.05, ***p*<0.01, ****p*<0.001. Additional controls variables not shown: Age, Gender and years of formal education. | | | | |

Table 5: Agency and Experience factor scores for human questions reward and punishment in each individual group.

|  | **Punishment** |  | **Reward** |  |
| --- | --- | --- | --- | --- |
|  | **β (SE)** |  | **β (SE)** |  |
| *Stu. Relig* |  |  |  |  |
| Intercept | 0.43 (1.76) | [-3.18, 4.04] | 0.01 (1.38) | [-2.97, 2.18] |
| Agency | 0.30 (0.13)* | [0.06, 0.51] | 0.63 (0.10)*** | [0.36, 0.79] |
| Experience | 0.05 (0.12) | [-0.18, 0.27] | -0.08 (0.09) | [-0.27, 0.08] |
| *Stu. Not Relig* | |  |  |  |
| Intercept | 0.15 (0.86) | [-1.44, 1.63] | -0.67 (0.73) | [-1.91, 0.63] |
| Agency | 0.39 (0.07)*** | [0.19. 0.57] | 0.79 (0.07)*** | [0.65, 0.89] |
| Experience | 0.24 (0.09)* | [0.03, 0.44] | -0.09 (0.08) | [-0.23, 0.05] |
| *Mtu. Relig* |  |  |  |  |
| Intercept | 0.25 (0.68) | [-1.03, 1.48) | -0.35 (0.49) | [-1.24, 0.60] |
| Agency | 0.47 (0.12)*** | [0.22, 0.68] | 0.86 (0.09)*** | [0.73, 1.05] |
| Experience | 0.28 (0.13)* | [0.06, 0.50] | 0.09 (0.09) | [-0.08, 0.28] |
| *Mt. Not Relig* |  |  |  |  |
| Intercept | 0.42 (0.49) | [0.60, 0.18] | -0.06 (0.51) | [-1.44, 0.96] |
| Agency | 0.45 (0.06)*** | [0.24, 0.62] | 0.68 (0.06)*** | [0.45, 0.84] |
| Experience | 0.36 (0.07)*** | [0.17, 0.57] | 0.13 (0.08)† | [-0.03, 0.35] |
| *Hindu* |  |  |  |  |
| Intercept | 0.48 (0.30) | [-0.11, 1.04] | -0.12 (0.17) | [-0.45, 0.21] |
| Agency | 0.05 (0.16) | [-0.31, 0.44] | 0.45 (0.09)*** | [0.15, 0.70] |
| Experience 1 | 0.45 (0.10)*** | [0.21, 0.60] | 0.05 (0.06) | [-0.07, 0.16] |
| Experience 2 | -0.35 (0.09)*** | [-0.52, 0.17] | 0.01 (0.05) | [-0.07, 0.07] |
| *Muslim* |  |  |  |  |
| Intercept | 0.64 (0.62) | [-0.49, 1.76] | -1.06 (0.35)** | [-2.03, -0.30] |
| Agency | -0.36 (0.24) | [-0.84, 0.07] | 1.12 (0.13)*** | [0.73, 1.41] |
| Experience 1 | 0.47 (0.17)** | [0.14, 0.82] | -0.12, 0.10) | [-0.27, 0.03] |
| Experience 2 | -0.36 (0.12)** | [-0.60, -0.10] | 0.03 (0.07) | [-0.07, 0.14] |
| *ITaukei Christ.* | |  |  |  |
| Intercept | -1.27 (0.62)* | [-2.73, 0.04] | 0.54 (0.29)† | [-0.07, 1.07] |
| Agency | 0.54 (0.36) | [-0.19, 1.37] | 0.43 (0.18)* | [0.08, 0.75] |
| Experience 1 | 1.00 (0.21)*** | [0.61, 1.34] | -0.22 (0.10)* | [-0.40. 0.006] |
| Experience 2 | 0.16 (0.19) | [-0.23, 0.56] | 0.16 (0.09)† | [-0.01, 0.35] |
| *Note:* †*p*<0.10, **p*<0.05, ***p*<0.01, ****p*<0.001. Additional controls variables not shown: Age, Gender and years of formal education. | | | | |

Table 6: Agency and Experience factor scores for God questions predicting reward and punishment in each individual group.

## Predicting reward and punishment using all North Americans (rather than only non-religious and Christians

|  | **Punishment** | | **Reward** | |
| --- | --- | --- | --- | --- |
|  | **β (SE)** | **[95% CI]** | **β (SE)** | **[95% CI]** |
| *North Americans and iTaukei Christians* | | | |  |
| Intercept | 0.20 (0.33) | [-0.46, 0.87] | -0.02 (0.26) | [-0.52, 0.47] |
| Agency | 0.46 (0.11)*** | [0.22, 0.66] | 0.86 (0.08)*** | [0.73, 1.03] |
| Experience | 0.28 (0.12)* | [0.06, 0.49] | 0.11 (0.09) | [-0.07, 0.27] |
| Mtu. Not Relig | 0.24 (0.15)† | [-0.03, 0.51] | -0.02 (0.11) | [-0.28, 0.21] |
| Stu. Relig | -0.27 (0.16)† | [-0.59, 0.03] | 0.03 (0.13) | [-0.22, 0.26] |
| Stu. Not Relig | -0.03 (0.15) | [-0.36, 0.26] | -0.01 (0.12) | [-0.22, 0.22] |
| iTa. Christ | -1.06 (0.25)*** | [-1.57, -0.54] | 0.28 (0.20) | [-0.03, 0.61] |
| A*Mtu. Not Rel. | -0.03 (0.14) | [-0.30, 0.27] | -0.18 (0.11)† | [-0.44, 0.03] |
| A*Stu. Rel. | -0.18 (0.16) | [-0.47, 0.15 | -0.22 (0.12)† | [-0.50, 0.00] |
| A*Stu. Not Rel. | -0.09 (0.14) | [-0.37, 0.20] | -0.08 (0.11) | [-0.28, 0.09] |
| A*iTa. Christ | -0.02 (0.36) | [-0.81, 0.67] | -0.45 (0.28) | [-0.84, -0.02] |
| E*Mtu. Not Rel. | 0.09 (0.15) | [-0.21, 0.40] | 0.04 (0.12) | [-0.20, 0.29] |
| E*Stu. Rel. | -0.23 (0.16) | [-0.53, 0.07] | -0.19 (0.12) | [-0.45, 0.05] |
| E*Stu. Not Rel. | -0.04 (0.15) | [-0.32, 0.26] | -0.20 (0.12) | [-0.46, 0.02] |
| E*iTa. Christ | 0.72 (0.19)*** | [0.33, 1.07] | -0.14 (0.15) | [-0.38, 0.10] |
| *Note:* †*p*<0.10, **p*<0.05, ***p*<0.01, ****p*<0.001. Additional controls variables not shown: Age, Gender and years of formal education. | | | | |

Table 7: Comparison between iTaukei Christians and all North Americans, rather then just Christian and non-religious.
